# Alcoholic Pancreatitis: Pathogenesis, Incidence and Treatment with Special Reference to the Associated Pain

**DOI:** 10.3390/ijerph6112763

**Published:** 2009-11-04

**Authors:** Raffaele Pezzilli, Antonio M. Morselli-Labate

**Affiliations:** Department of Digestive Diseases and Internal Medicine, Sant’ Orsola-Malpighi Hospital, Via Massarenti 9, 40138 Bologna, Italy; E-Mail: antonio.morselli@unibo.it

**Keywords:** alcoholic pancreatitis, pain, gene modification, histology, inflammation, patghogenesis, endoscopy, surgery

## Abstract

Alcoholic pancreatitis continues to stir up controversy. One of the most debated points is whether from onset it is a chronic disease or whether it progresses to a chronic form after repeated episodes of acute pancreatitis. Histological studies on patients with alcoholic pancreatitis have shown that the disease is chronic from onset and that alcoholic acute pancreatitis occurs in a pancreas already damaged by chronic lesions. Genetic factors may also play a role in the pathogenesis of alcoholic disease. The incidence of chronic alcoholic pancreatitis seems to have decreased in the last twenty years. Finally, recent therapeutic studies which have shown medical or surgical approaches capable of reducing the pain episodes in chronic pancreatitis patients will be described.

## 1. Introduction

Alcoholic pancreatitis as well as chronic pancreatitis of other etiologies is clinically characterized by frequent painful episodes in the early stages of the disease and then, over time, the pain attacks decrease in frequency and intensity in parallel with the progressive destruction of the gland. The pain tends to disappear during the natural course of the disease (the so-called “burn-out” phenomenon) or after a period of time (ranging from a few years to a maximum of 10 years from the onset of the disease), and the destruction of the gland is clinically evident with the appearance of steatorrea and diabetes [1]. Pain significantly decreases the quality of life of chronic pancreatitis patients and sometimes leads to severe malnutrition [2]. The typical profile of a patient with chronic pancreatitis is that of a drinking male of about 40 years of age, with moderate pain intensity located in the epigastrium and radiating to the back, independent of food intake and lasting less than one day [3]. Of course, there is a small percentage of patients who have persistent abdominal pain and this group of subjects represents a therapeutic challenge. On the other hand, in some patients, the disease may occur without pain (so-called painless pancreatitis) and the disease becomes clinically evident with the appearance of complications; this is the case of the major part of patients having autoimmune pancreatitis [4] and in a low percentage of patients with alcoholic pancreatitis. The most frequent complications of chronic pancreatitis are the following: obstructive jaundice, duodenal stenosis, left-sided portal hypertension, and pseudocyst and mass formation; pancreatic carcinoma may also occur as a complication of chronic pancreatitis [5]. In this paper we will review the epidemiological, pathological and clinical aspects of chronic pancreatitis as well as the recent advances in pancreatic pain treatment based on the papers appeared in the last decade.

## 2. Epidemiology

In 1998, Lankisch and Banks reported that the prevalence of chronic pancreatitis in many parts of the world appeared to be in the range of 3–10 per 100,000 people [6]. Most cases of chronic pancreatitis require hospitalization due to the presence of pain as well as to the appearance of other complications.

**Figure 1.**
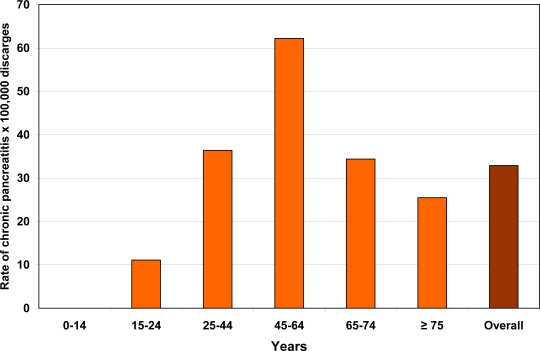
Rate of patients discharged from the Italian hospitals in the year 2005 having the diagnosis of chronic pancreatitis and stratified according to age.

**Figure 2.**
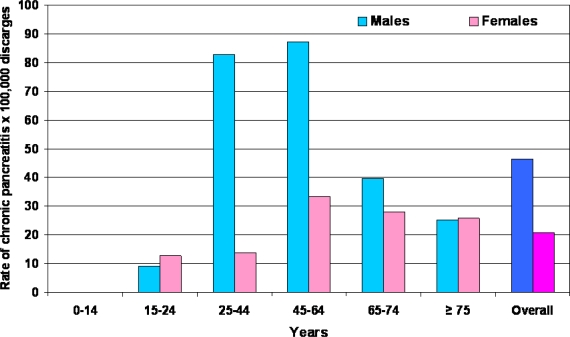
Rate of patients discharged from the Italian hospitals in the year 2005 having the diagnosis of chronic pancreatitis and stratified according to gender and age.

**Figure 3.**
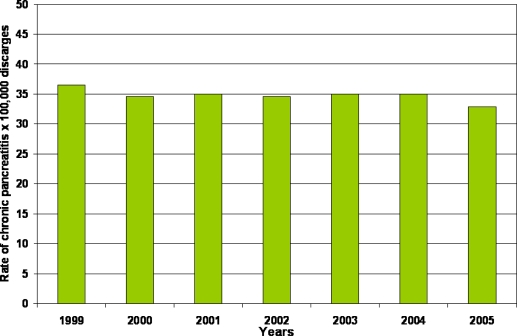
Trend in the rate of patients discharged from the Italian hospitals from 1999 to 2005 having a diagnosis of chronic pancreatitis.

In Italy, according to the data reported by the Department of the Welfare in 2005 [7], the rate of patients discharged for chronic pancreatitis is 32.9 per 100,000 hospitalized patients (Figure 1). The age of the major part of these patients ranges from 45 to 64 years (Figure 1) and the majority are males (Figure 2). The mean length of hospitalization is 9.8 days and this figure is higher as compared to that of all patients with diseases other than chronic pancreatitis (7.5 days). Finally, in Italy the trend of hospitalization for chronic pancreatitis seems to have decreased from 1999 to 2005 (Figure 3). There is no doubt that, in Western countries, alcohol is the most frequently associated factor of chronic pancreatitis, chronic alcoholic pancreatitis presents mainly in young adults of 30–40 years of age, and the prevalence is higher in the male gender. In these countries, in the period from 1940 to 2003, alcohol frequency increased as an etiological factor of chronic pancreatitis from 19 [8] to 50% [9] and even up to 80% [10–12]. The results of the latter study regarding the etiology of chronic pancreatitis were subsequently confirmed by others in Europe [13–19] as well as in Brazil [20], Australia [21] and South Africa [22].

On the other hand, four consecutive surveys carried out in Japan (from 1970 to 1977, from 1977 to 1984, in 1994, and in 1999, respectively) [23] showed that alcohol as an etiological factor accounted for less than 60% and this figure is similar among the various periods studied.

## 3. The Importance of Alcohol as an Etiological Factor

From a practical point of view, understanding the pathogenesis may lead to the identification of novel molecular targets and the development of new potential therapeutic agents. Thus, the role of alcohol is the cornerstone of the pathogenesis of chronic pancreatitis. Durbec and Sarles [11] have clearly demonstrated that alcohol is a risk factor for chronic pancreatitis; in fact, they showed that the relative risk would be multiplied approximately by a factor of 1.4 per 20-gram increase in alcoholic intake. Furthermore, the risk appears to be more evident when passing from the class of non-drinkers to that of a 20-gram alcohol intake per day (relative risk of about 2); these data have been subsequently confirmed by others [24].

## 4. Changing Lifestyle Modifies the Alcoholic Etiology of Chronic Pancreatitis

The impact of a changing lifestyle, especially in developing countries, may contribute to modifying the etiology of chronic pancreatitis. For example, alcohol consumption in developing countries has increased [25] and this has changed the etiology of chronic pancreatitis in those countries. On the contrary, in Europe, there was a progressive reduction of alcohol consumption from 1961 to 1991 [26] and the alcoholic form seems to have been reduced in frequency [3]. Furthermore, taking into account the lifestyle of chronic pancreatitis patients, it has been reported that the pancreatic functional changes caused by alcoholic pancreatitis progress even after the cessation of alcohol use, but the progression is slower and less severe when alcohol intake is stopped [27].

## 5. Genetic Factors

The possibility of evaluating the mutations of the cystic fibrosis transmembrane conductance regulator-gene (CFTR-gene), as well as the discoveries of mutations of both the cationic trypsinogen gene (protease-serine-1 gene, PRSS-1) and the serine protease inhibitor, Kazal type 1 gene (SPINK-1), have led to better evaluating the hereditary forms in Western countries.

In particular, Le Bodic *et al.* [28] found a link between hereditary pancreatitis and a locus on the long arm of chromosome 7 (7q33-qter), and Whitcomb *et al.* [29] better defined this finding, restricting the region of interest to 7q35. Whitcomb *et al.* [30] have also reported the identification of a mutation of the gene for cationic trypsinogen (PRSS1) in this region as the factor responsible for hereditary pancreatitis in the five families they studied. This mutation results in the modification of the cationic trypsinogen molecule, specifically the substitution of the arginine in position 122 with histidine. The authors suggested that this mutation could cause interference with the self-limiting mechanisms of cationic trypsinogen activation which, in the case of intraparenchymal activation of the enzyme, could determine an overactivation of the enzyme, with the consequent development of acute pancreatitis.

Another gene examined in studying of chronic pancreatitis is the pancreatic secretory trypsin inhibitor (SPINK1). Witt *et al.* [31] found the modification of this gene in 22 of 96 young patients with chronic pancreatitis (28 hereditary pancreatitis, 68 idiopathic). The most significant mutation resulted in the substitution of asparagine in position 34 with serine. This mutation caused a loss in the efficacy of the inhibition of trypsinogen activation. Mutations in the pancreatic secretory trypsin inhibitor (SPINK 1) are present in 20% of subjects with idiopathic, in 5% of those with alcoholic chronic pancreatitis and in 50% of those with tropical pancreatitis; thus this gene is not able to determine chronic pancreatitis by itself, but seems capable of predisposing the disease in the presence of other precipitating factors [32].

Mutations of CFTR may also play a role in the development of alcoholic pancreatitis. We studied mutations of CFTR in 98 patients of whom 34 had acute pancreatitis (20 of biliary origin, 14 alcoholic), 46 had chronic pancreatitis (34 of alcoholic origin, one biliary, one autoimmune, 10 idiopathic) and 18 had pancreatic cancer [33]. We found an elevated prevalence (17.4%) of CFTR mutations in the group of patients with chronic alcoholic pancreatitis and concluded that such mutations most likely have a predisposing role for the disease.

Finally, Verlaan *et al.* [34] studied the potential role of the polymorphisms of the ADH3 and CYP2E1 genes codifying the alcohol-metabolizing enzymes in 142 adult patients with chronic pancreatitis (21 hereditary pancreatitis, 82 alcoholic origin, and 39 idiopathic) and found no evidence of association between these genes and pancreatitis.

In conclusion, the PRSS1 mutations appear capable of inducing chronic pancreatitis whereas CFTR and SPINK-1 seem to be “gene modifiers” capable of inducing the disease in the presence of a risk factor such as alcohol.

## 6. Modification of the Etiology

The changing lifestyle and the discovery of genetic factors associated with pancreatitis may contribute to changing the frequencies of the various etiologies of chronic pancreatitis. This is the reason why, from 2004 to the present, the etiological features of chronic pancreatitis have been reported to be different than in the past. Four studies are examples of this. In Korea [35], the main etiological factor remains alcohol (64.3%), followed by an unknown etiology (20.8%), obstruction (8.6%) and autoimmune pancreatitis (2.0%). In a recent survey on chronic pancreatitis in the Asian-Pacific region [36], there was a great variability in the frequency of alcoholic pancreatitis, accounting for about 19% of chronic pancreatitis cases in China up to 95% in Australia whereas tropical pancreatitis was 46.4% in China and was, obviously, not present in Australia. In a recent survey of chronic pancreatitis in Italy [3], chronic pancreatitis associated with alcohol abuse accounted for less than 50% of cases and this figure is lower than that reported by Gullo *et al.* in 1977 [10]. However, some regional differences regarding the frequency of alcoholic chronic pancreatitis exist in Italy. In fact, in Bologna (located in Northern Italy), alcohol as an etiological factor remains high (80.4%) [37] whereas, in Sicily (located in the Southern Italy), the percentage of alcoholic chronic pancreatitis is about 60% [38]. In a survey of chronic pancreatitis in Italy 3], alcohol as an etiological factor of chronic pancreatitis is followed by obstruction (27%), pancreatitis of unknown origin (17%), autoimmunity (4%) and hereditary/genetic factors (4%). The most surprising results come from India. In a prospective nationwide study in India [39], the authors found that the majority of patients had pancreatitis of unknown origin (60% of the cases); alcoholic chronic pancreatitis accounted for a third of the cases whereas tropical pancreatitis was present in only 3.8% of the cases. It seems that alcohol tends to be increasing in frequency in India as is chronic pancreatitis of unknown etiology; the increase in the idiopathic form of the disease (60% of all forms of chronic pancreatitis are idiopathic) has not been explained well by the authors and may be due to an incomplete evaluation of the possible risk factors. In this regard, it is worth noting that the frequency of chronic pancreatitis of unknown origin is 17% in the Italian survey [3] ranging from about 12% in Bologna to 38% in Sicily [38,39]. Thus, we need to spend more time with our patients when evaluating the possible factors determining their disease.

## 7. Pathogenesis of Alcoholic Pancreatitis

The mechanism which determines the main manifestation of chronic pancreatitis, i.e., fibrosis of the pancreatic gland, has been well summarized by Taludkar *et al.* [40]. In brief, the oxidation of ethanol to acetaldehyde determines the activation of the pancreatic stellate cells in the quiescent state without any pre-activation; this process generates a state of oxidant stress within the pancreatic stellate cells which subsequently activates the downstream pathways of the fibrogenesis. This finding implies that, in the human pancreas, pancreatic stellate cells may be stimulated early during chronic alcohol intake even in the absence of necro-inflammation. The importance of the oxidative stress in chronic pancreatitis patients has also been reported using breath analysis [41]. In this study, H_2_S, NO and a substance having a molecular mass of 66 u (M66) were those which had significantly higher breath concentrations in chronic pancreatitis patients than in healthy subjects after adjustment for the ambient air; no significant differences in H_2_S, M66, and NO were found between patients with and without alcoholic pancreatitis.

Regarding the pancreatic stellate cells, in 1982, Watari *et al.* [42] reported the presence of vitamin A-containing cells in the vitamin A-fed rat pancreas. These were later described and characterized as stellate cells in the rat and the human pancreas [43,44]. Pancreatic stellate cells are morphologically similar to hepatic stellate cells. They have long cytoplasmic processes and are situated close to the pancreatic acini. In the quiescent state, these cells contain lipid droplets, store vitamin A and express markers such as desmin, glial fibrillary acidic protein, neural cell adhesion molecule and neurotrophin nerve growth factor just as hepatic stellate cells do. Pancreatic stellate cells contain enzyme alcohol dehydrogenase [45] and, when activated, they assume a myofibroblast-like phenotype [46]. Activated pancreatic stellate cells are characterized by the disappearance of fat globules and the expression of alpha-smooth muscle actin. These cells have proliferative and migratory [47–49] functions, and they also synthesize and secrete extracellular fibrous tissue matrix proteins, matrix metalloproteinases and their inhibitors [50]; it has also been demonstrated that pancreatic stellate cells have phagocytic activity [51]. Thus, the ability of pancreatic stellate cells to synthesize as well as to degrade extracellular matrix proteins suggests their role in maintaining a normal pancreatic architecture which can shift towards fibrogenesis if the balance is altered. Ethanol, acetaldehyde and oxidant stress are capable of activating pancreatic stellate cells via three mitogen-activated protein kinase pathways [52], namely extracellular signal kinase, p38 kinase and c-jun amino terminal kinase [53–55], and ethanol and acetaldehyde are also capable of activating phosphatidylinositol 3-kinase and protein kinase C [56]. On the other hand, extracellular signal kinase activation occurs via a signal transduction pathway which involves G-protein Ras and serine threonine protein kinase Raf-1 [57,58]. The Ras superfamily G proteins undergo post-translational modification involving isoprenylation, a process which requires intermediate substrates of cholesterol biosynthesis [59,60] which is regulated by HMG CoA reductase [61]. The paracrine pro-fibrogenic effect of TGF-beta on pancreatic stellate cells is mediated via Smad while the autocrine effect is mediated through the extracellular signal kinase pathway [62]; furthermore, the role of the peroxisome proliferator-activated receptor-gamma seems to be involved in the activation of pancreatic stellate cells [63,64].

The major parts of the studies published on pancreatic stellate cells have been carried out on experimental animals; thus, the study of Suda *et al.* seems to be of particular interest because it was carried out on humans [65]. These authors investigated the distribution of activated pancreatic stellate cells or myofibroblasts using immunohistochemistry and a computer-counting device in relation to fibrogenesis in 24 patients with clinically diagnosed chronic alcoholic pancreatitis. In all cases, fibrosis was patchily distributed in the perilobular or interlobular areas accompanied by a cirrhosis-like appearance; it had extended into the intralobular area in advanced cases. Seven patients had a massive or confluent loss of exocrine tissue, resulting in extensive interlobular fibrosis; the more extensive the interlobular fibrosis, the smaller the lobules. Immunoreactivity to alpha-smooth muscle actin, a myofibroblast marker, was found mostly in the same areas of the fibrosis, mainly the interlobular and less often the periacinar, areas; the average percentage area of perilobular myofibroblasts was significantly higher than that of periacinar myofibroblasts in 20 randomly selected lobules; the fibrosis also immunostained positive for collagen types I and III. In conclusion, this study, carried out on humans, further supports the hypothesis that the fibrotic alterations in chronic alcoholic pancreatitis are not due to recurrent episodes of necrotizing pancreatitis but the disease is due to a chronic stimulation of alcohol on pancreatic stellate cells which play an important role in pancreatic fibrogenesis.

## 8. Histology of Alcoholic Pancreatitis

**Figure 4.**
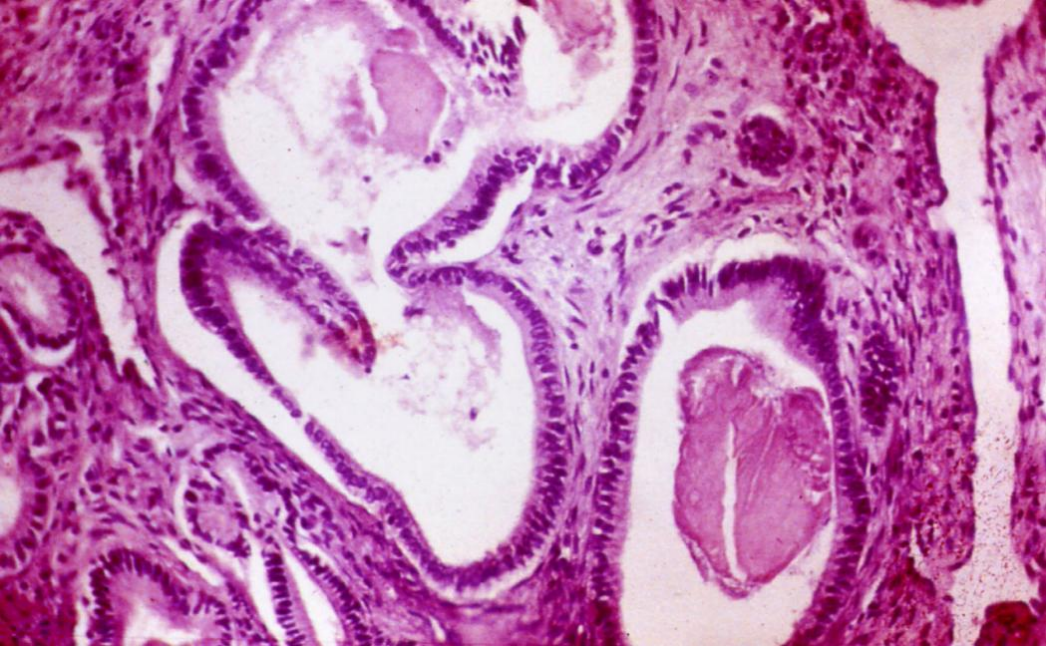
Typical pathological appearance of human chronic pancreatitis: Fibrosis, loss of acinar tissue, enlarged ducts and stones within some of them.

According to the previously described pathogenesis, alcohol seems to induce pancreatic fibrosis (Figure 4) as it has frequently been found in autoptic series of alcoholics without a clinical history of chronic pancreatitis [66–68]. Two histological studies [69,70] were conducted on the pancreata of patients at the time of their first attack of acute alcoholic pancreatitis, and both studies found the presence of chronic lesions in the acutely inflamed pancreas. Moreover, in a clinical study [71] of 114 patients hospitalized for acute alcoholic pancreatitis as the first manifestation of pancreatic disease, a diagnosis of chronic pancreatitis was made in 105 (92%) of the 114 patients during the follow-up. These authors concluded that acute alcoholic pancreatitis without underlying chronic pancreatitis does not exist or is extremely rare.

Based on the data of the literature and on our experience, we believe that the great majority (>90%) of alcoholic patients who present clinically with acute pancreatitis also have chronic lesions. The possibility that an alcoholic with acute pancreatitis has no chronic pancreatic lesions certainly cannot be excluded but, if this condition exists, it is rare [72].

A working hypothesis called ‘the necrosis-fibrosis sequence’ has been also suggested for explaining the pathogenesis of chronic pancreatitis. This hypothesis is based on pancreatic tissue examination during alcoholic acute and chronic pancreatitis [73–75]. Early after the onset on pancreatitis symptoms there is evidence of postnecrotic changes such as pseudocystes; and morphology from surgical specimens showing predominant pancreatic lesions of focal necrosis and mild fibrosis. In contrast, specimens several years after the onset of symptoms obtained at autopsy show severe perilobular and intralobular fibrosis and calcification with no necrosis [76].

## 9. Alcohol and Pancreatic Inflammation

Singh *et al.* [77] in an effort to develop a model of chronic alcoholic pancreatitis in rats fed a nutritionally adequate diet, three groups of animals each were fed ad libitum, Lieber-DeCarli diet with 40% of carbohydrate calories replaced by ethanol ad libitum and isocaloric amounts of Lieber-DeCarli diet respectively for a period of 18 months. In these animals basal and secretin-stimulated pancreatic juice was obtained as well as pancreatic tissue for evaluating the presence of histological, biochemical analyses and cellular analyses. All of the ethanol-fed animals developed morphological changes akin to human chronic pancreatitis. In the pancreatic tissue of animals fed ethanol, total protein, trypsinogen and free trypsin were increased and amylase was decreased. While acid phosphatase was increased in all of the particulate fractions, cathepsin B was increased in the zymogen granule and mitochondrial-lysosomal fractions. Basal and post-secretin pancreatic juice did not show a significant increase in digestive or lysosomal enzymes. Thus, the authors concluded that focal degenerative changes may be due to trypsin generated by intracellular activation of digestive enzymes by lysosomal enzyme cathepsin B.

Gukovsky *et al.* [78] have recently found that rats which had been fed ethanol for 8 weeks and which had received cyclosporin A for the last two weeks and in which acute pancreatitis had been cerulein-induced had a massive loss of acinar cells, persistent inflammatory infiltration and fibrosis as compared to the control animals receiving cyclosporin A plus cerulein. Furthermore, macrophages in the treated rats were prominent in the inflammatory infiltrate and showed a marked increase in pancreatic NF-kappaB activation, cytokine/chemokine mRNA expression, collagen and fibronectin, in the expression and activities of matrix metalloproteinases 2 and 9 and in the activation of pancreatic stellate cells. Therefore, this study shows the possible mechanism by which alcohol sensitizes the pancreas to chronic injury.

## 10. Alcohol and Pancreatic Pain

The close relationship between inflammation and pain has been investigated by Michalski *et al.* [79]. They found that the responsiveness of peripheral blood mononuclear cells to the neuropeptide pituitary adenylate cyclase-activating polypeptide is altered in chronic pancreatitis patients. In fact, the nociceptive status of chronic pancreatitis patients correlated with pancreatic pituitary adenylate cyclase-activating polypeptide levels and with IL-10 bias of pituitary adenylate cyclase-activating polypeptide-exposed peripheral blood mononuclear cells of chronic pancreatitis patients. Thus, the ability of peripheral blood mononuclear cells to produce and to respond to pituitary adenylate cyclase-activating polypeptide might influence the neuroimmune interactions which regulate pain and inflammation in chronic pancreatitis.

The chronic inflammatory process produces a massive release of local lytic enzymes which leads to the release of several pro-inflammatory cytokines and pro-fibrotic agents, such as TGF-beta1 and growth factor B derived from platelets and capable of inducing fibrosis. As a result of this process, there is also a mechanism of neurogenic inflammation mediated by transient receptor potential vanilloid-1 (TRPV-1) [80,81]. Other receptors involved in this process are the T-type calcium channels (located on sensory intrapancreatic fibers) activated by hydrogen sulfide. The activation of peripheral nerve fibers determines the synthesis of substance P; the synthesis of substance P is also increased by protein 43 which is overexpressed in chronic alcoholic pancreatitis. Substance P carries out many activities [82]: it stimulates inflammatory cells to produce cytokines through the neurokinin 1 receptor, it increases poly-morphonuclear cells, macrophages and fibroblasts locally and induces nerve regeneration, and it also causes a vasoconstriction of arterioles determining ischemia of the nervous fibers themselves. Moreover, pain in chronic pancreatitis is the result of the effect of somatic and visceral fibers which result in changes in neurons [83]. This phenomenon leads to a hyperalgesia mediated by the central nervous system even in the absence of a peripheral nociceptive stimulus. This mechanism is associated with a functional rearrangement of the cerebral cortex which leads to a “pain memory” over time and is independent of the peripheral input [36].

## 11. Other Causes of Pain in Chronic Pancreatitis

Regarding the pancreatic causes of pain, recent studies [84,85] have shown a neuropathic origin of this symptom, i.e. normal pain signals in the presence of injury or disease of the peripheral and/or central nervous system. This is supported by Drewes *et al.* [86] who evaluated electroencephalography in chronic pancreatitis patients having pain. In these subjects, an increase in theta wave activity was found and this seemed to be a marker of neuropathic pain. Furthermore, it is also known that, in the pancreatic tissue of patients with chronic pancreatitis, there is an increase in the number and diameter of nerve fibers [86]. We also note the extrapancreatic causes of pain are mainly related to duodenal stenosis associated or not with stenosis of the main biliary duct [87].

## 12. Role of Alcoholic Chronic Pancreatitis in Pancreatic Cancer

Several studies have linked chronic pancreatitis with an increased risk of pancreatic cancer [88]. The evidence comes from different types of studies, with case-control studies being the most frequent. Most of these studies have shown that when compared to control subjects without chronic pancreatitis, patients with chronic pancreatitis have an increased risk of pancreatic cancer. However, these studies are subject to recall bias because patients with pancreatic cancer may be more likely to report a past history of pancreatitis than control subjects. Furthermore, because chronic pancreatitis is a rare disease, a large number of patients are necessary to be sure that a negative study is truly negative rather than being “non-significant” as a result of the small sample size. Thus, cohort studies provide more reliable evidence of the link between chronic pancreatitis and pancreatic cancer. Several such studies have been carried out and all show an elevated risk of pancreatic cancer, even after excluding patients in whom there has been a short interval between the onset of pancreatitis and cancer [89].

## 13. A New Perspective for the Treatment of Intractable Pain in Chronic Alcoholic Pancreatitis Patients

Regarding the therapeutic modalities in curing chronic pancreatitis patients, all studies enroll all types of pancreatitis and there are no specific studies only for the alcoholic form. The persistence of pancreatic pain leads to surgery in 11% of patients and to endoscopic treatment in approximately 29% of the cases [3]. After surgical treatment, the disappearance of pain, or at least its substantial reduction, is observed in 67–94% in the short term whereas good results are achieved in 50–88% of the subjects over a long-term period [36]. Thus, we need adequate medical treatment, especially for those patients who do not benefit from surgical or endoscopic approaches. The traditional medical approach is based on the administration of long-acting octreotide [90], tricyclic antidepressants, narcotics or a celiac plexus nerve block using computed tomography or echoendoscopy, and the results are sometimes disappointing. For this reason, studies exploring additional techniques for alleviating pain in chronic pancreatitis patients are needed.

### 13.1. Control of the Central Nervous System

Kongkam *et al.* have evaluated the efficacy of the intrathecal narcotics pump [91]. They studied 13 patients who had experienced intractable upper abdominal pain from chronic pancreatitis. Each patient had had other unsuccessful treatment modalities, including partial pancreatic resections in six patients. They were offered an intrathecal narcotics pump after a successful intraspinal opioid trial. The etiology of chronic pancreatitis was unknown in three subjects, due to cystic fibrosis in two and due to alcohol abuse in two; pancreas divisum was present in the remaining six patients. The median duration of severe, intractable pain prior to the intrathecal narcotics pump was six years and the median follow-up time after the intrathecal narcotics pump was 29 months. The intrathecal narcotics pump was in situ for a mean duration of 29 months. Seven patients had a pump exchange or removal for various reasons, such as improvement of pain, meningitis, meningitis with subsequent replacement or pump failure. The mean pain score prior to implantation was significantly higher than that calculated one year after entry into the study. The median oral narcotic dose before and one year after the intrathecal narcotics pump were morphine sulfate equivalents of 337.5 mg/day and 40 mg/day, respectively (P < 0.01). Two patients were considered failures as they still required a high dosage of both oral and intrathecal medications to control their pain, despite significant pain-score improvement; one patient who was excluded due to meningitis was also considered a failure. Therefore, the overall success rate of the intrathecal narcotics pump based on an intention-to-treat analysis was 76.9%. The major complications of the intrathecal narcotics pump observed in 3 patients (23.1%) were central nervous system infection requiring pump removal, cerebrospinal fluid leak requiring laminectomy and perispinal abscess with bacterial meningitis requiring pump removal. This study shows a good efficacy of the intrathecal narcotics pump in chronic pancreatitis patients with persistent pain; however, as previously reported [92], the risk of this therapeutic modality is high. Furthermore, we need longer follow-ups and especially therapeutic trials comparing pancreatectomy, the intrathecal narcotics pump and implanted nerve stimulators.

### 13.2. Antoxidants

Antioxidant treatment may become a useful tool in preventing and curing pain in patients with chronic pancreatitis. Data on this topic are under debate [93] because the majority of studies published are not randomized or are based on a low number of patients. Recently a placebo-controlled double blind trial has reported good results on pain relief using antioxidant supplementation on a large number of chronic pancreatitis patients [94]. In this study, consecutive patients with chronic pancreatitis were randomized to groups which were given placebo or antioxidants for six months. The primary outcome measure was pain relief; secondary outcome measures were analgesic requirements, hospitalization, and markers of oxidative stress and antioxidant status. One hundred and twenty-seven patients (86 male, 35 alcoholic, and 92 with idiopathic chronic pancreatitis) were studied; fifty-six patients were assigned to the placebo group and 71 to the antioxidant group. After six months, the reduction in the number of painful days per month was significantly higher in the antioxidant group as compared to the placebo group. The reduction in the number of analgesic tablets per month was also higher in the antioxidant group. Furthermore, 32% and 13% of patients became pain free in the antioxidant and placebo groups, respectively. Thus, the results of this study seem to confirm that antioxidant supplementation is effective in relieving pain and reducing levels of oxidative stress in patients with chronic pancreatitis. However, we need studies confirming these promising results and comparing this treatment to others.

### 13.3. Surgical or Endoscopic Approach?

Many papers have been published on the possibilities offered by interventional endoscopy but, on the other hand, several studies have also been produced on the positive results offered by surgery. However, there are very few studies comparing the two procedures. In 2003, Dite *et al.* [95] published the results of a prospective, randomized trial comparing endoscopic and surgical therapy for chronic pancreatitis. Seventy-two patients were randomized; surgery consisted of resection and drainage procedures while endotherapy included sphincterotomy and stenting and/or stone removal In the entire group, the initial success rates were similar for both groups but, at the 5-year follow-up, the complete absence of pain was more frequent after surgery (37% vs. 14%), with both procedures having a similar rate of partial relief (49% vs. 51%). The authors concluded that surgery is superior to endotherapy for long-term pain reduction in patients with painful obstructive chronic pancreatitis. They also suggested that a better selection of patients for endotherapy could be helpful in maximizing results. Furthermore, due to its low degree of invasiveness, endotherapy can be offered as a first-line treatment, with surgery being performed in case of failure and/or recurrence. The results of this study were received with skepticism by endoscopists, mainly because the treatment was tailored to the patient whereas surgery involved resection in the majority of patients; endoscopic drainage techniques were not optimally applied and shock-wave lithotripsy was not utilized. Several other papers utilizing sphincterotomy, dilation of strictures, and the removal of stones found endoscopic therapy to be beneficial in patients with chronic pancreatitis [96,97]. More recently, a new comparative study on the long term results of endoscopic vs. surgical treatment of chronic pancreatitis patients was published [98].The authors randomized 39 symptomatic patients having chronic pancreatitis with distal obstruction of the pancreatic duct and without an inflammatory mass: 19 underwent endoscopic transampullary drainage (16 of whom also underwent lithotripsy) and 20 had operative pancreaticojejunostomy. The primary end point was the average Izbicki pain score during 2 years of follow-up. The secondary end points were pain relief at the end of follow-up, physical and mental health, morbidity, mortality, length of hospital stay, number of procedures undergone and changes in pancreatic function. During the 24 months of follow-up, patients who underwent surgery, as compared with those who were treated endoscopically, had significantly lower Izbicki pain scores and significantly better physical health summary scores evaluated using the Medical Outcomes Study 36-Item Short-Form General Health Survey questionnaire. At the end of the follow-up, complete or partial pain relief was achieved in 32% of patients assigned to endoscopic drainage as compared to 75% of patients assigned to surgical drainage; this difference was statistically significant. Complication rates, length of hospital stay and changes in pancreatic function were similar in the two treatment groups, but patients receiving endoscopic treatment required more procedures than the patients in the surgical group (a median of eight vs. three, P < 0.001). Once again, surgical drainage of the pancreatic duct was more effective than endoscopic treatment in patients with obstruction of the pancreatic duct due to chronic pancreatitis. What can we learn from these results? Is surgery the best approach to chronic pancreatitis patients? In the experimental condition evaluated by Cahen *et al.* [98], the answer is ‘yes’.

## 14. Conclusions

The data discussed above show that alcoholic pancreatitis is chronic its onset. Acute pancreatitis in an alcoholic is generally secondary to chronic pancreatitis. Determinants of individual susceptibility to alcoholic pancreatitis still remain uncertain. However, studies of the fibrogenesis and genetic modifications of alcoholic pancreatitis have improved our knowledge of this disease, demonstrating that SPIN-1 and CFTR gene modification may act as modifier genes. The studies in progress lead us to a clear understanding of the mechanisms involved in the pathogenesis of alcoholic pancreatitis. Antioxidants may be useful in treating patients with recurrent pain; in the case of intractable pain, an intrathecal narcotics pump may be offered. In those patients in whom medical therapy failed to obtain persistent pain relief, a surgical approach should be preferred over an endoscopic approach.
